# Psychological effects of anti-Arab politics on American and Arab peoples’ views of each other

**DOI:** 10.1371/journal.pone.0301282

**Published:** 2024-05-01

**Authors:** Youngki Hong, Angela T. Maitner, Kyle G. Ratner

**Affiliations:** 1 Department of Psychology, Columbia University, New York, NY, United States of America; 2 Department of Psychological and Brain Sciences, University of California, Santa Barbara, Santa Barbara, CA, United States of America; 3 Department of Psychology, American University of Sharjah, Sharjah, United Arab Emirates; Carnegie Mellon University, UNITED STATES

## Abstract

Disparaging rhetoric about Arab people was prevalent during Donald Trump’s political rise in the United States. Although this rhetoric was intended to energize conservative Americans, it also echoed throughout many liberal parts of the United States and around the world. In this research, we experimentally examined the effects of such rhetoric on American and Arab people’s attitudes and visual representations of each other before and after Trump was elected. Although people overwhelmingly reported not liking the negative rhetoric, the rhetoric alone did not influence explicit and implicit intergroup biases in either location, as measured by feeling thermometers and Implicit Association Tests. However, the election outcome moderated the way rhetoric influenced how American and Arab people visually represented each other. Our research sheds light on nuanced effects of global politics on various information processing stages within intergroup perception.

## Introduction

Analysis of Donald Trump’s rise in American politics often focused on effects of his incendiary rhetoric on his supporters. His election correlated with an uptick in the perceived social acceptance of expressing prejudice and even hate crime [1–3]. Although Trump directed his rhetoric toward his base, the coverage of his rallies and social media posts reverberated nationwide and globally [4]. In his initial presidential campaign, Trump made controversial remarks about Muslim and Arab people. Exploiting stereotypes of Middle Eastern individuals as terrorists, Trump’s messaging struck a chord with a segment of the U.S. population shaped by conflicts in the region, terrorist incidents, and negative media portrayals of Arab Muslims [5, 6]. When Trump won the 2016 Presidential election, he quickly imposed restrictive immigration policies, including a travel ban, often referred to as the “Muslim Ban”, that restricted admission of citizens from seven predominantly Muslim countries (Exec. Order No. 13769, 2017).

The current research examined social cognitive effects of the 2016 U.S. Presidential election and anti-Arab rhetoric on individuals in the U.S. (California) and in an Arab country (the United Arab Emirates). We specifically sought to examine the following questions: What were the psychological effects of being exposed to anti-Arab political rhetoric on American individuals who did not necessarily agree with it? How did people in the Arab world react to the same rhetoric that was targeting them? Did it matter whether politicians using such rhetoric were aligned with the views of the Presidential administration that had the power to carry it into action? Our work sought to address these questions through a comparative analysis across different regions, consideration of real-world politics, and experimentation, providing insight into the nuanced psychological effects of politics at a global scale.

People often assume that their attitudes and behaviors are impervious to outside influences [7]. Ideologically liberal Americans, for instance, might believe that their attitudes about various social groups were immune to Trump’s views about these groups. However, the extant literature on intergroup bias suggest that people’s attitudes do not always match their egalitarian values [8–10] because passive exposure to cultural stereotypes can mentally associate certain groups with negative attributes [8]. Indeed, relevant to the current investigation, recent studies showed that even Democrats avoid groups negatively portrayed by former President Trump [11]. Concerningly, Arab individuals are often negatively represented in U.S. news media [5], and experimental research demonstrates that exposure to anti-Muslim information can impact Americans’ attitudes [6].

The idea that an individual’s attitudes assimilate toward cultural stereotypes is consistent with foundational social cognition research on priming and concept accessibility [12, 13]. Recent work has argued that regional differences in social biases can be explained by one’s environment (e.g., what appears on television and what people around you think) influencing concept accessibility [14]. From this vantage, it is possible that exposure to anti-Arab rhetoric may activate negative associations about the Arab world in American culture in American people’s minds and their attitudes would shift accordingly. Similarly, exposure to anti-Arab rhetoric could make stereotypes about Americans as racist and hostile toward Arab individuals more accessible in Arab people’s minds. In short, anti-Arab rhetoric could simultaneously activate negative associations of Arab people in the minds of American people and negative associations of American people in the minds of Arab people, leading to more negative attitudes toward and perceptions of each other.

Despite considerable evidence for situationally triggered concept accessibility effects on attitudes and beliefs, it would be overly simplistic to characterize people consuming political rhetoric as purely passive information processors. As the classic “mindlessness” research points out, people can think carefully when they are motivated to do so [15]. Other research on motivated reasoning finds that people are less willing to accept information that they do not want to believe [16, 17]. From this vantage, exposure to anti-Arab rhetoric could lead to an asymmetric change in attitudes and perception when comparing responses of American and Arab people. That is, liberal Americans may disregard or even contrast away from anti-Arab politics because it goes against their ideological commitments but people in the Arab world may similarly disregard the rhetoric or express more negative views of Americans because anti-Arab politics threatens their wellbeing.

Although the political vitriol expressed by Trump and like-minded politicians toward the Arab World and Muslims seems like it would be psychologically impactful on American and Arab people, it is important to note that “American” and “Arab” are not novel attitude objects and thus attitudes toward them are difficult to change. Decades of conflict have solidified attitudes in both regions, potentially rendering them resistant to change. Research on implicit attitudes indicates difficulties in shifting well-formed attitudes [18]. Crandall and colleagues (2018) found that Trump influenced beliefs in prejudice’s acceptability, but self-reported prejudicial attitudes did not change [1]. Similarly, exposure to anti-Arab rhetoric may not prompt American participants to adopt rhetoric-congruent attitudes. Arab people’s longstanding views about Americans might not also change because of exposure to anti-Arab rhetoric. This leads to a prediction that in response to anti-Arab rhetoric, American people’s attitudes and evaluations of Arab people would stay the same or become more positive, whereas Arab people’s attitudes and evaluations of American people would stay the same or become more negative.

Given that intergroup bias is not a monolith and leaks out during different stages of information processing, it is possible that different predictions can be made about the effects of rhetoric on different stages of information processing. According to Gilbert et al. (1988), the first step when encountering social information, such as political rhetoric, is categorization. This involves figuring out what you are seeing and bringing to mind the prototypical representations that define the social categories to make sense of the world [19]. For instance, Americans exposed to right wing political rhetoric might think about stereotypes about Arab people because information associated with infamous terrorist attacks on the United States homeland are activated. This same rhetoric could bring to mind in Arab people stereotypes about American politicians who espouse such rhetoric and thus this dominates their mental image of the prototypical American. Face categorization tasks optimized for the reverse correlation image classification technique have been utilized by social psychologists for over a decade to estimate the prototypical mental representations that perceivers associate with a social category [20–22]. The reverse methods have been useful for understanding visual representations of social categories, including racial groups [21]. In fact, the methods have revealed visual representations that Americans have of Arab people [23] and can easily be adapted to examine how Arab people visually represent Americans.

After categorization, automatic associations can drive attitudes. Characterization in Gilbert’s model is defined by how people spontaneously associate certain attributes with different attitude objects [19]. For example, what are the attributes that Arab people associate with American people and how are such associations influenced by the exposure to anti-Arab rhetoric? To assess this stage of information processing, we included an indirect measure of attitudes, specifically the Implicit Association Test (IAT) [24, 25], that reflects behavioral responses that are efficient and not easy to control. If people have the opportunity to control their responses, then they can adjust their characterization [19]. Because people may be able to regulate the expression of bias on direct measures of attitudes [8], we included feeling thermometers, which are face-valid and not time-constrained, reflecting individuals’ expression of attitudes that they desire to share with the world.

Inclusion of a measure of visual representation as well as explicit and implicit measures of attitudes allowed us to examine whether there are any dissociations between more effortless, implicit processing, such as visual representation and IAT, and more effortful, explicit processing, such as responses to feeling thermometers. For example, it is possible that liberal Americans could be swayed by anti-Arab rhetoric on an implicit-level due to concept accessibility effects even if they ultimately reject the rhetoric at an explicit level, leading to more prejudiced visual representation of and negative implicit attitudes towards, yet steady or even more positive explicit attitudes toward Arab people. On the other hand, Arab people might perceive anti-Arab rhetoric as a threat to their country’s honor [26] and show elevated levels of bias on both explicit and implicit measures, if they are not resistant to change at all.

### Research overview

In this research, we experimentally presented a news article to American participants living in a predominantly politically liberal part of the United States and Arab participants living in the Arab region (a group that was frequently targeted by right-wing American politicians). In the key experimental condition, the news article quoted an American politician disparaging Arab people as “dangerous terrorists” and “untrustworthy”. We also included other between-subjects experimental conditions that exposed participants to either a content matched pro-Arab news article or no political rhetoric. We then used reverse correlation, the IAT, and feeling thermometers to assess the effects of these rhetoric conditions on how the American and Arab participants viewed each other. Our experimental manipulation was further nested within a naturalistic quasi-experiment, in that we conducted our research in two identical waves—before Trump became President and then after he was elected and rapidly enacted anti-Muslim policies. This pre/post design allowed us to investigate the relationship between Trump holding Presidential authority and how people in the United States and the Arab region viewed each other.

## Materials and method

The study was conducted pre- and post-election at two sites: A university in California (USA Site) and a university in the United Arab Emirates (UAE Site). Participants were randomly assigned to one of three conditions: (1) pro-Arab rhetoric, (2) anti-Arab rhetoric, or (3) no rhetoric condition. All data, analysis scripts, and study materials are posted at https://figshare.com/s/dc35f47a9edc3ef483a3. In all data collection, responses were made anonymously, and we were not able to identify individual participants during or after data collection. All studies were reviewed and approved by the Institutional Review Board (IRB) of University of California Santa Barbara (USA Site) and American University of Sharjah (UAE Site).

### Wave 1a: Americans’ views of Arabs before the election/travel ban

Before the 2016 Presidential election in the United States (May 12 –October 20, 2016), 212 American students from a university in California (M_age_ = 19.13, SD = 1.47; 144 female, 62 male, and 6 unidentified) were recruited to participate in a study about America and the Arab World in exchange for course credit. This sample and all subsequent samples of participants provided written consent. The racial and ethnic breakdown of our sample was 79 White, 67 Asian, 27 Latinx, 6 Black, 2 Pacific Islander/ Hawaiian, 1 Arab, 10 multiracial, and 19 other. Participants were told that they would perform several tasks that may include a simple memory task involving reading a short article, several categorization tasks with different words and pairs of faces, and answering several questions about themselves. Next, participants were randomly assigned to one of three conditions: (1) pro-Arab rhetoric, (2) anti-Arab rhetoric, or (3) no rhetoric condition.

In the pro-Arab rhetoric condition, participants (n_1_ = 71) first read a news article about an American politician discussing recent terrorist attacks in the United States. In this article, he argues that the problem is *not* the Arab World and calls for *decreased* U.S. military presence in Arab countries and *allowing* people from Arab countries to feel welcome in the U.S. to help build trust. In the anti-Arab rhetoric condition, participants (n_2_ = 71) read a similar news article about an American politician discussing recent terrorist attacks in the United States. In this article, however, the politician argues that the problem *is* the Arab World and calls for *increased* U.S. military presence in Arab countries and *preventing* people from Arab countries from entering the U.S. because they might be terrorists. In both conditions, participants were given one minute to read the article and were asked three questions regarding the news article (e.g., what is the name of the politician in the article?) as an attention check. Participants then completed two tasks: the IAT [24, 25] and a face categorization task optimized for a reverse correlation analysis (the task order was randomized across participants.) In the no rhetoric condition, participants (n_3_ = 70) completed the implicit association test and the face categorization task without reading any news articles.

**Fig 1:**
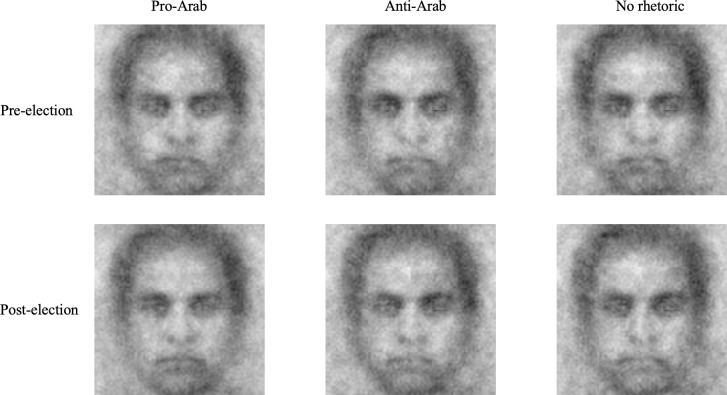
Group-level classification images of Arab faces (Time X condition).

The face categorization task optimized for reverse correlation analysis consisted of 300 trials. On each trial, participants selected an Arab face out of two adjacent grayscale face images. We used the grayscale neutral male average face of the Averaged Karolinska Directed Emotional Faces Database [27] as the base image to generate 300 pairs of face stimuli used in the face categorization task. Different noise patterns consisting of 4,092 superimposed truncated sinusoid patches were added to the same base image, generating 300 different noise patterns that each look unique [20]. A noise pattern was applied to the base image, and the inverse of that noise pattern was added to the base image, creating a pair of images. We presented inverse noise faces equally on the left and right sides of the screen in a random order. We used the same pairs of faces for all participants. Next, we carried out reverse correlation image classification by using the R package, *rcicr* [28] and generated visual renderings of our participants’ representations of Arab faces. We did so by averaging noise patterns of the chosen 300 faces from the face categorization task for each participant and superimposing the normalized average noise pattern back onto the original base image to create participant-level classification images. After creating participant-level classification images, we created group-level classification images for all three conditions by averaging noise patterns of participant-level classification images in each condition and superimposing the normalized average noise pattern back onto the base image (Fig 1).

During the IAT, participants categorized words presented in the middle of the screen into one of four category labels that were presented on the top left corner or top right corner of the screen (two categories on each side). When the word belonged to a category on the left, participants pressed the E key, whereas when the word belonged to a category on the right, they pressed the I key. The words used in this IAT were identified as either American or Arab (American–Chicago, Florida, Los Angeles, New York, and Texas; Arab–Abu Dhabi, Egypt, Lebanon, Morocco, and Saudi Arabia) or were positive and negative words (positive–Good, Happy, Joy, Love, and Nice; negative–Bad, Evil, Hurt, Nasty, and Pain). In some blocks, the American category label was paired with the positive word category label on the same side of the screen and the Arab category label was paired with the negative word category on the opposite side of the screen. This way, participants had to press the same key when they saw words belonging to either the American or Positive word category, and the other key for words belonging to either the Arab or Negative word category. Because we expected American participants to respond faster in this type of blocks, we called these blocks “congruent” blocks. In other blocks, this combination was flipped; the American category label was paired with the negative word category label on the same side of the screen and the Arab category label was paired with the positive category label on the other side of the screen. Because we expected American participants to respond slower in this type of blocks, we called these blocks “incongruent” blocks. After practice blocks, participants completed each block type twice (20 trials in the first block and 40 trials in the second block). We computed IAT scores following Greenwald et al. (2003) [25]. Conceptually, the IAT scores represent the implicit preference for ingroup (American) over outgroup (Arab) by measuring how fast participants accurately responded in congruent blocks relative to incongruent blocks.

Lastly, participants completed various questionnaires including their reactions to the news article (for pro-Arab and anti-Arab rhetoric conditions only). These include their agreement with the statements made in the article on a scale of 1 (strongly disagree) to 5 (strongly agree), their impression of the politician in the article on a scale of 1 (very negative) to 5 (very positive), and emotional reactions after reading the article (how much they felt anger, satisfied, fear, pride, disgust, happiness, anxious, grateful guilt, respect, shame, irritated, and sadness) [29] on a scale of 1 (not at all) to 7 (extremely), political orientations on a scale of 1 (very liberal) to 7 (very conservative), and explicit ratings of American people and Arab people on a scale of 0 (extremely unfavorable) to 100 (extremely favorable). We computed an explicit ingroup bias by subtracting each participant’s feeling thermometer rating of Arab people from their feeling thermometer rating of American people.

After all the experimental sessions were completed, we created group-level reverse correlation classification images of what our participants in each condition thought that an Arab person looked like. We then assessed the objective differences in these images to examine how different rhetoric conditions would influence trait impressions of Arab faces. To do this, we had independent samples of raters who were not aware of the face generation stage rate three group-level Arab face classification images (pro-rhetoric, anti-rhetoric, and no rhetoric). Fifty-four participants were recruited through the TurkPrime website (www.turkprime.com) to complete an online survey administered through Qualtrics (www.qualtrics.com). After providing informed consent, participants rated the three group-level classification images on thirteen trait dimensions (i.e., To what extent is this face… trustworthy, attractive, dominant, caring, sociable, confident, emotionally stable, responsible, intelligent, aggressive, mean, weird, and unhappy?) [30]. We presented each face by itself in a random order, and the participants made ratings on scales from 1 (not at all) to 7 (extremely). The order of the trait presentation was also random. Expected completion time was 10 minutes, but participants had 30 minutes to complete the task. We compensated them with $1 for their participation.

### Wave 1b: Arabs’ view of Americans before the election/travel ban

Before the 2016 Presidential election in the U.S. (October 2 –October 10, 2016), 164 students at a university in the United Arab Emirates (M_age_ = 20.23, SD = 1.27; 99 female, 56 male, and 9 unidentified) were recruited to participate in a study about America and the Arab World in exchange for course credit. Because we recruited only Arab participants, we report their nationality instead of race and ethnicity: 46 Egyptian, 20 Emirati, 14 Syrian, 12 Iraqi, 11 Jordanian, 11 Palestinian, 8 Lebanese, 7 American, 4 Sudanese, 3 Canadian, 3 Saudi, 3 Tunisian, 2 Yemeni, and 20 other. Wave 1b followed a largely identical procedure as Wave 1a. The condition breakdown of participants was 54 pro-Arab rhetoric, 54 anti-Arab rhetoric, and 56 no rhetoric participants.

**Fig 2:**
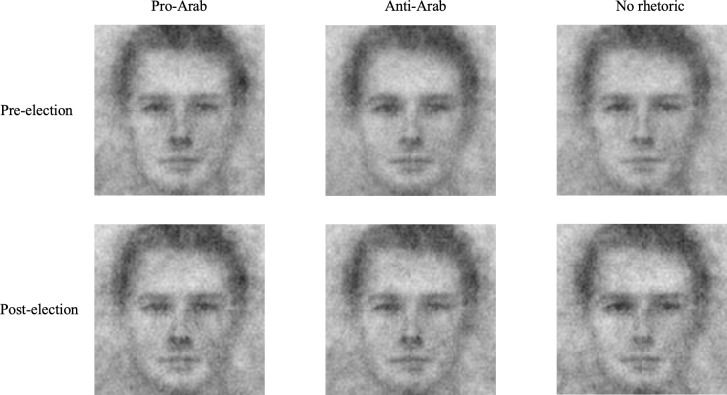
Group-level classification images of American faces (Time X condition).

There were two major ways Wave 1b differed from Wave 1a. First, we computed the IAT and explicit attitude scores differently. In the IAT, we expected Arab participants to respond faster in blocks where the Arab category and the positive word category were paired and the America category and the negative word category were paired (“congruent” blocks). On the other hand, we expected them to respond slower in blocks where the Arab category and the negative word category were paired and the America category and the positive word category were paired (“incongruent” blocks). Thus, by measuring how fast participants accurately responded in congruent blocks relative to incongruent blocks, the IAT scores in this study represented the implicit preference for ingroup (Arab) over outgroup (American). We also computed an explicit ingroup bias by subtracting each participant’s feeling thermometer rating of American people from their feeling thermometer rating of Arab people. Second, in the face categorization task participants were asked to select an American face on each trial instead of an Arab face. Therefore, we created three group-level reverse correlation classification images of American faces (pro-Arab rhetoric, anti-Arab rhetoric, and no rhetoric; Fig 2). We then assessed the objective differences in these images by asking an independent sample of 49 raters from TurkPrime to rate the three group-level classification images of American faces on the same thirteen trait dimensions as Wave 1a.

### Wave 2a: Americans’ views of Arabs after the election/travel ban

After the 2016 Presidential election in the U.S. (February 22 –May 12, 2017; after Trump’s widely publicized executive order banning travel into the United States from seven Muslim-majority countries on January 27, 2017), 223 American students from a university in California (M_age_ = 19.45, SD = 1.63; 144 female, 70 male, and 9 unidentified) were recruited in exchange for course credit. The racial and ethnic breakdown of our sample was 64 White, 62 Asian, 40 Latinx, 8 Black, 25 multiracial, 2 Pacific Islander/ Hawaiian, 3 Arab, and 19 other. The condition breakdown of participants was 72 pro-Arab rhetoric, 74 anti-Arab rhetoric, and 77 no rhetoric participants. 53 independent participants rated the group-level classification images.

### Wave 2b: Arabs’ views of Americans after the election/travel ban

After the 2016 Presidential election in the U.S. and the travel ban executive order (February 6 –March 9, 2017), 169 students from a university in the United Arab Emirates (M_age_ = 19.97, SD = 1.45; 105 female, 59 male, and 5 unidentified) were recruited in exchange for course credit. The nationality breakdown of this sample was 36 Egyptian, 27 Emirati, 21 Syrian, 18 Iraqi, 17 Palestinian, 14 Jordanian, 7 Lebanese, 7 Saudi, 4 American, 4 Sudanese, 2 Bahraini, and 12 other. The condition breakdown of participants was 55 pro-Arab rhetoric, 57 anti-Arab rhetoric, and 57 no rhetoric participants. 50 independent participants rated the group-level classification images.

## Results

### Reactions to the news article

We first examined how Rhetoric Type (pro-Arab, anti-Arab), Site (UAE, USA), and Time (pre-, post-election) and interactions among these variables influenced whether participants agreed with the news article, their impression of the politician depicted in the news article, and their emotional reactions to the news article.

#### Agreement with the news article

We conducted a three-way analysis of variance with condition (pro-Arab, anti-Arab rhetoric), site (UAE, USA), time (pre-, post-election), and all possible interactions among those three variables (condition X site, condition X time, site X time, condition X site X time) as factors predicting participants’ agreement with the article. The results showed a main effect of condition, F(1, 446) = 773.91, p < .001, ηp2 = .63 indicating that on average participants agreed more with the pro-Arab (M = 3.94, SD = .89) than the anti-Arab (M = 1.66, SD = .88) rhetoric article. The main effect of site was not significant, F(1, 446) = .15, p = .698, ηp2 < .001, neither was the main effect of time, F(1, 446) = .03, p = .867, ηp2 < .001. The only significant interaction effect was between condition and site, F (1, 446) = 16.36, p < .001, ηp2 = .03. A follow-up post-hoc test of this interaction effect using Tukey’s HSD revealed that Arab participants agreed with the pro-Arab rhetoric (M = 4.11, SD = .91) more than American participants (M = 3.80, SD = .85), p = .046. On the other hand, American participants agreed with the anti-Arab rhetoric (M = 1.83, SD = .92) more than Arab participants (M = 1.47, SD = .79), p = .011.

#### Impression of the politician

We conducted a three-way analysis of variance with condition (pro-Arab, anti-Arab rhetoric), site (UAE, USA), time (pre-, post-election), and all possible interactions among those three variables (condition X site, condition X time, site X time, condition X site X time) as factors predicting participants’ impression of the politician in the article. The results showed a main effect of condition, F(1, 448) = 898.15, p < .001, ηp2 = .67 indicating that on average participants had a more favorable impression of the politician in the pro-Arab (M = 3.74, SD = .83) than the anti-Arab (M = 1.57, SD = .72) rhetoric article. The main effect of site was not significant, F(1, 448) = 1.19, p = .276, ηp2 = .003, neither was the main effect of time, F (1, 448) = .16, p = .690, ηp2 < .001. The only significant interaction effect was between condition and site, F (1, 448) = 8.44, p = .004, ηp2 = .02. A follow-up post-hoc test of this interaction effect using Tukey’s HSD revealed that American participants had a more favorable impression of the politician in the anti-Arab rhetoric article (M = 1.70, SD = .82) than Arab participants (M = 1.41, SD = .55), p = .026.

#### Emotional reactions to the news article

We used a repeated-measures multivariate analysis of variance (rMANOVA) to test the effects of condition, site, time and the interaction among them on participants’ emotional reactions to the news article. Significant multivariate effects were found for condition (Pillai’s Trace = .56, F = 33.37, df = (13, 340), p < .001), site (Pillai’s Trace = .30, F = 11.06, df = (13, 340), p < .001), and condition X site interaction (Pillai’s Trace = .13, F = 3.97, df = (13, 340), p < .001). Thus, we only used condition, site, and the interaction between the two for the univariate analyses of individual emotions. The univariate F tests showed that all emotional reactions (except for guilt) to pro-Arab and anti-Arab rhetoric were significantly different from each other at the .05 significance level. Specifically, participants felt more angry, anxious, disgusted, fearful, irritated, sad, and shameful, and felt less grateful, happy, pride, respect, and satisfaction after reading the anti-Arab rhetoric article compared to the pro-Arab rhetoric article. Significant main effects of site were also found for disgust, grateful, guilt, happiness, pride, respect, satisfaction, and shame: American participants felt less disgust, guilt, happiness, pride, respect, and satisfaction, and felt more shame than Arab participants. Interaction effects were found for some emotional reactions including anxious, grateful, happiness, pride, respect, and satisfaction. The univariate F test results including the means, standard deviations, F values, p values, and effect sizes (comparing pro-Arab and anti-Arab conditions within American and Arab samples) for each emotional reaction are presented in Table 1.

**Table 1:** Emotional reactions to the news article 2 X 2 rANOVA results.

	USA			UAE			F-values		
	Pro-Arab (SD)	Anti-Arab (SD)	Cohen’s d	Pro-Arab (SD)	Anti-Arab (SD)	Cohen’s d	Condition	Site	Interaction
Anger	1.41 (1.61)	3.55 (2.08)	1.15	1.47 (1.70)	3.87 (2.17)	1.22	112.48***	.76	.01
Anxious	1.31 (1.46)	2.18 (1.85)	.52	1.41 (1.95)	1.44 (1.71)	.02	5.06*	2.38	5.71*
Disgust	1.51 (1.87)	3.88 (2.37)	1.10	1.73 (2.17)	4.83 (2.25)	1.40	119.58***	7.84**	1.96
Fear	1.07 (1.38)	2.23 (1.89)	.69	.92 (1.56)	1.70 (1.92)	.44	25.11***	3.29^+^	.2.15
Grateful	1.47 (1.83)	.66 (1.29)	.51	3.20 (2.46)	.95 (2.02)	.99	60.64***	19.94***	17.20***
Guilt	1.79 (1.89)	1.81 (1.87)	.01	.68 (1.22)	.67 (1.34)	.01	.01	37.19***	.02
Happiness	1.40 (1.77)	.33 (.73)	.79	2.97 (2.43)	.20 (.62)	1.50	127.49***	22.47***	31.71***
Irritated	1.72 (2.17)	3.99 (2.09)	1.07	1.83 (2.01)	4.39 (2.33)	1.17	92.47***	1.23	.00
Pride	1.27 (1.58)	.56 (1.08)	.53	2.73 (2.41)	1.02 (2.01)	.76	45.277***	22.94***	11.78***
Respect	2.66 (2.25)	.51 (1.09)	1.22	4.46 (2.27)	.32 (1.02)	2.26	267.95***	17.73***	34.79***
Sadness	2.11 (2.20)	3.50 (2.39)	.60	2.17 (2.26)	3.54 (2.46)	.58	24.71***	.15	.26
Satisfaction	2.39 (1.94)	.66 (1.22)	1.06	3.71 (2.19)	.35 (.78)	1.98	201.47***	11.95***	26.69***
Shame	1.84 (1.97)	3.16 (2.27)	.62	.89 (1.45)	1.90 (2.28)	.53	28.95***	22.96***	.69

Significance codes

*** < .001

** < .01

* < .05 ^+^ < .10

In brief, participants generally agreed more with the content of and liked the politician better in the pro-Arab versus anti-Arab article. Furthermore, participants at both sites felt more negative emotions after reading the Anti-Arab rhetoric article compared to the Pro-Arab rhetoric article.

### Visual representations

Reverse correlation image classification uses responses on a face categorization task to estimate a perceiver’s mental image of a certain social category. We thus used a face categorization task optimized for a reverse correlation image classification analysis to examine our American participants’ prototypical representation of Arabs and our Arabs participants’ prototypical representation of Americans. Following reverse correlation convention [19], we then had an independent sample rate these representations to objectively quantify them. Because American participants and Arab participants received different prompts for the face categorization task (i.e., “which looks more Arab?” for American participants and “which looks more American?” for the UAE-based participants), we conducted two separate mixed-design multivariate analyses of variance on the ratings of the images. Rhetoric Type (within-subjects factor; pro-Arab, anti-Arab, no rhetoric), Time (between-subjects; pre-, post-election), and the Rhetoric Type × Time interaction served as factors in our analyses. These analyses were followed by a univariate analysis of variance for each trait rating. We show the results below separated by site.

#### Visual representations of Arab people (Site: USA)

We found significant multivariate effects for all variables: Rhetoric Type (Pillai’s Trace = .49, F = 5.02, df = (26,398), p < .0001), Time (Pillai’s Trace = .39, F = 4.63, df = (13,93), p < .0001), and Rhetoric Type X Time (Pillai’s Trace = .58, F = 6.19, df = (26,398), p < .0001). The univariate F test results including the means, standard deviations, F values, p values, and effect sizes (comparing pro-Arab, anti-Arab, and no rhetoric conditions within pre- and post-election) for each trait are presented in Table 2.

**Table 2:** Trait rating 2X3 mixed design ANOVA results–USA.

	Pre-election				Post-election				F-values		
	Pro-Arab (SD)	Anti-Arab (SD)	No rhetoric (SD)	η^2^_condition_	Pro-Arab (SD)	Anti-Arab (SD)	No rhetoric (SD)	η^2^_condition_	Condition	Time	Condition X Time
Aggressive	5.26 (.26)	4.85 (.23)	4.96 (.25)	.01	4.85 (.27)	4.45 (.22)	5.13 (.28)	.02	2.93^+^	.58	1.53
Attractive	3.52 (.20)	3.15 (.16)	3.44 (.18)	.01	4.06 (.23)	3.98 (.23)	3.49 (.20)	.02	3.51*	3.64^+^	5.21**
Caring	3.72 (.20)	3.65 (.22)	3.94 (.20)	.01	4.36 (.27)	3.77 (.20)	3.72 (.21)	.03	1.99	.55	3.42*
Confident	4.52 (.22)	4.63 (.22)	4.46 (.24)	.00	5.00 (.24)	5.11 (.25)	4.51 (.26)	.02	3.96*	1.32	1.62
Dominant	5.09 (.25)	5.44 (.26)	5.02 (.25)	,01	4.30 (.21)	5.21 (.28)	5.00 (.27)	.04^@^	8.14***	1.28	3.29*
Emotionally Stable	4.43 (.20)	4.00 (.20)	4.33 (.21)	.01	5.11 (.25)	4.45 (.24)	4.26 (.21)	.04^#^	7.77***	1.87	3.38*
Intelligent	4.54 (.21)	4.30 (.20)	4.57 (.21)	.01	5.09 (.24)	4.77 (.22)	3.64 (.19)	.14^#^^$^	16.60***	.02	23.42***
Mean	4.35 (.23)	5.07 (.24)	5.07 (.25)	.04	4.83 (.25)	5.02 (.27)	5.06 (.26)	.00	4.60*	.22	1.40
Responsible	4.65 (.21)	4.33 (.20)	4.59 (.21)	.01	4.26 (.21)	4.15 (.19)	4.62 (.24)	.02	5.03**	.46	1.61
Sociable	3.98 (.19)	3.43 (.17)	3.65 (.19)	.03	4.40 (.25)	3.89 (.23)	3.64 (.20)	.04^#^	8.52***	1.53	1.47
Trustworthy	4.07 (.22)	3.67 (.19)	4.07 (.20)	.02	4.64 (.27)	4.04 (.22)	3.89 (.20)	.04	5.99**	.99	3.35*
Unhappy	5.76 (.23)	4.31 (.19)	5.35 (.25)	.12^@^^#^	5.25 (.26)	5.92 (.24)	6.00 (.28)	.03	5.76**	4.16*	19.66***
Weird	5.02 (.26)	5.61 (.25)	5.09 (.26)	.02	3.55 (.24)	3.96 (.26)	4.40 (.28)	.03	6.59**	15.87***	5.44**

Tukey HSD significance results codes (p < .05)

^@^ pro-Arab ≠ anti-Arab

^#^pro-Arab ≠ no rhetoric

^$^anti-Arab ≠ no rhetoric

Significance codes

*** < .001

** < .01

* < .05 ^+^ < .10

#### Visual representations of American people (Site: UAE)

We found significant multivariate effects for all variables: Rhetoric Type (Pillai’s Trace = .54, F = 5.25, df = (26,366), p < .0001), Time (Pillai’s Trace = .36, F = 3.62, df = (13,85), p = .0002), and Rhetoric Type X Time (Pillai’s Trace = .42, F = 3.76, df = (26,366), p < .0001). The univariate F test results including the means, standard deviations, F values, p values, and effect sizes (comparing pro-Arab, anti-Arab, and no rhetoric conditions within pre-election and post-election) for each trait rating are presented in Table 3.

**Table 3:** Trait rating 2X3 mixed design ANOVA results–UAE.

	Pre-election				Post-election				F-values		
	Pro-Arab (SD)	Anti-Arab (SD)	No rhetoric (SD)	η^2^_condition_	Pro-Arab (SD)	Anti-Arab (SD)	No rhetoric (SD)	η^2^_condition_	Condition	Time	Condition X Time
Aggressive	4.45 (1.50)	4.65 (1.56)	4.92 (1.57)	.02	4.52 (1.49)	5.02 (1.49)	4.54 (1.62)	.02	2.78^+^	.01	2.99^+^
Attractive	5.22 (1.26)	5.22 (1.25)	5.18 (1.29)	.00	4.62 (1.46)	5.08 (1.51)	5.08 (1.64)	.02	2.16	1.35	2.52^+^
Caring	4.94 (1.42)	5.10 (1.45)	4.82 (1.24)	.01	5.08 (1.55)	4.94 (1.73)	5.02 (1.39)	.00	.26	.07	.81
Confident	5.90 (1.37)	5.69 (1.26)	5.55 (1.42)	.01	5.46 (1.59)	5.76 (1.42)	5.26 (1.55)	.02	3.53*	.80	1.96
Dominant	5.18 (1.44)	5.10 (1.26)	5.63 (1.42)	.03	4.92 (1.54)	5.48 (1.63)	5.10 (1.40)	.02	2.62^+^	.34	5.31**
Emotionally Stable	4.65 (1.38)	5.31 (1.54)	4.51 (1.36)	.06^$^	5.48 (1.66)	5.36 (1.60)	5.14 (1.50)	.01	5.45**	4.16*	3.45*
Intelligent	5.73 (1.29)	4.86 (1.10)	4.55 (1.26)	.15^@^^#^	5.68 (1.38)	5.46 (1.39)	5.56 (1.36)	.00	13.47***	6.09*	8.02***
Mean	4.20 (1.65)	4.37 (1.59)	4.86 (1.62)	.03	4.40 (1.74)	4.96 (1.64)	4.76 (1.45)	.02	5.57**	.73	2.46^+^
Responsible	4.84 (1.07)	4.67 (1.11)	4.71 (1.08)	.00	5.60 (1.48)	5.22 (1.47)	5.38 (1.38)	.01	2.35^+^	9.69**	.36
Sociable	5.29 (1.43)	5.16 (1.50)	4.00 (1.21)	.15^#^^$^	5.28 (1.74)	4.92 (1.65)	4.78 (1.43)	.02	15.35***	.55	5.55**
Trustworthy	5.59 (1.21)	4.41 (1.29)	5.14 (1.19)	.14^@^^$^	5.38 (1.50)	5.32 (1.67)	4.94 (1.57)	.02	10.71***	.49	11.06***
Unhappy	4.86 (1.53)	5.12 (1.60)	5.27 (1.58)	.01	4.54 (1.64)	5.20 (1.68)	5.16 (1.63)	.03	4.99**	.21	.60
Weird	3.98 (1.73)	3.86 (1.74)	3.96 (1.88)	.00	4.26 (1.96)	4.26 (1.87)	4.42 (1.87)	.00	.44	1.30	.22

Tukey HSD significance results codes (p < .05)

^@^ pro-Arab ≠ anti-Arab

^#^pro-Arab ≠ no rhetoric

^$^anti-Arab ≠ no rhetoric

Significance codes

*** < .001

** < .01

* < .05 ^+^ < .1

### Representational similarity analysis

The no rhetoric condition provides a window into participants’ default representation of the other group (i.e., what they naturally think the other group looks like when not experimentally exposed to political rhetoric). Multiple regression representational similarity analysis (RSA) [31, 32] allowed us to examine how similar or dissimilar the default representation of an outgroup face (either Arab or American) is to when people are exposed to the pro-Arab rhetoric versus the anti-Arab rhetoric. We also examined whether Trump becoming President and enacting anti-Muslim policies was related to the shifts in similarity structures of these representations. Simply put, did our participants’ default mental representation of the outgroup resemble more their mental representation of the outgroup when exposed to anti-Arab or pro-Arab? Moreover, did these effects differ before and after the election?

**Fig 3:**
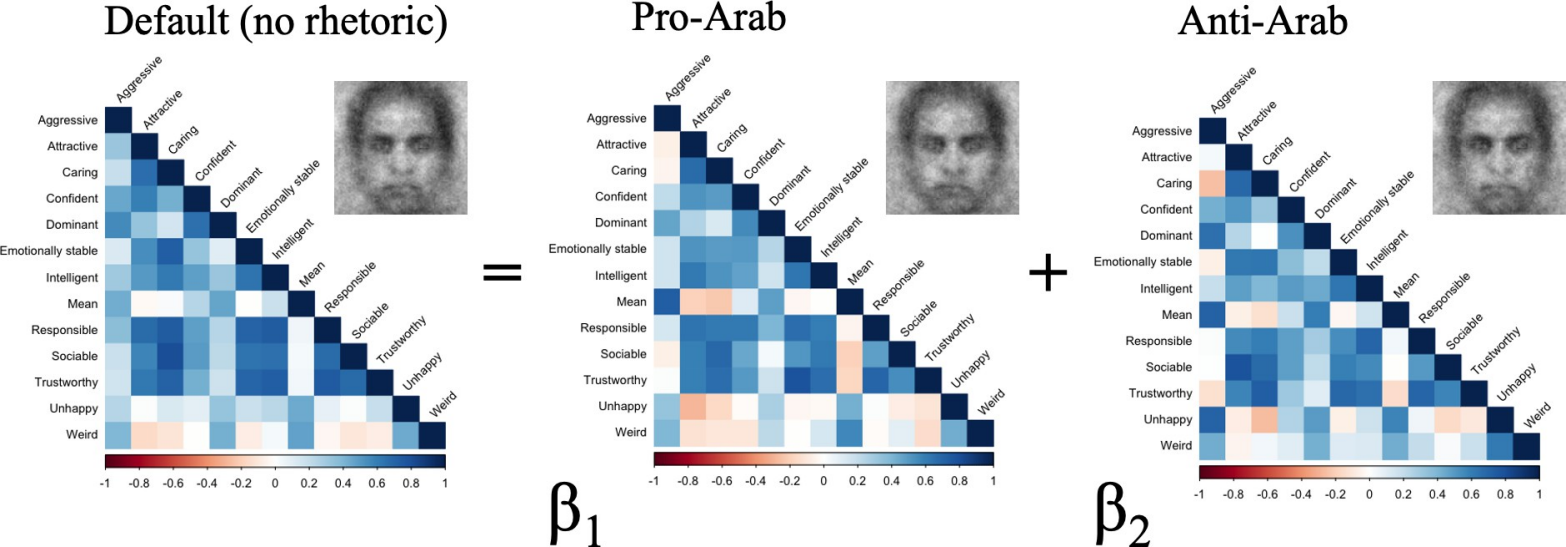
Multiple regression RSA illustration.

To quantitatively examine similarities between default trait representations of outgroup face images and trait representations of outgroup face images after exposure to different political rhetoric, we first computed pairwise correlations of trait rating data, generating a correlation matrix for each group-level classification image. We then vectorized unique pairwise correlation matrices (i.e., excluding duplicate correlation coefficients). Finally, we predicted vectors of default (i.e., no rhetoric) trait rating data with linear combinations of vectors of appropriate pro-Arab and anti-Arab trait rating data. For example, we predicted unique pairwise correlation coefficients (i.e., trait representations) of the pre-election Arab face in the no rhetoric condition using the linear combination of trait representations of pre-election Arab faces in the pro-Arab and anti-Arab rhetoric conditions (Fig 3). In other words, we tested similarities of pro-Arab and anti-Arab rhetoric outgroup face images to default outgroup face images while controlling for each other by using multiple regression RSA.

#### Arab representational similarity as a function of rhetoric (Site: USA)

We found that *before* the election, the pro-Arab condition trait representation was a significant predictor of the no rhetoric condition trait representation (β = .747, SE = .101, t = 6.53, p < .001), whereas the anti-Arab condition trait representation was not significant (β = .170, SE = .105, t = 1.48, p = .142). Linear hypothesis testing revealed that the pro-Arab condition trait representation predicted the no rhetoric condition trait representation significantly better than the anti-Arab condition trait representation, F(1,75) = 6.39, p = .014). That is, our American participants’ default representation of an Arab person before Trump became President was similar to the representation they generated when they heard an American politician speak positively about the Arab World. This is consistent with an overall positive mental image of Arabs.

We found that *after* the election, the pro-Arab condition trait representation remained a significant predictor of the no rhetoric condition trait representation (β = .690, SE = .103, t = 5.23, p< .001) and the anti-Arab condition trait representation remained a non-significant predictor (β = .219, SE = .110, t = 1.66, p = .102). However, linear hypothesis testing showed that the pro-Arab condition trait representation and the anti-Arab condition trait representation did not significantly differ in their predictability of the no rhetoric condition trait representation, F(1,75) = 2.92, p = .092.

#### American representational similarity as a function of rhetoric (Site: UAE)

Again, we used ordinary least squares multiple regression to predict trait representations of the pre-election, no rhetoric condition American faces with the linear combination of the trait representations of pre-election, pro-Arab condition American faces and trait representations of pre-election, anti-Arab condition American faces. We found that *before* the election both the pro-Arab condition trait representation (β = .541, SE = .117, t = 3.83, p < .001) and the anti-Arab condition trait representation (β = .310, SE = .125, t = 2.19, p = .031) were significant predictors of the no rhetoric condition trait representation. Linear hypothesis testing showed that the pro-Arab condition trait representation and the anti-Arab condition trait representation did not significantly differ in how much they predicted the no rhetoric condition trait representation (F(1,75) = 0.55, p = .460).

**Fig 4:**
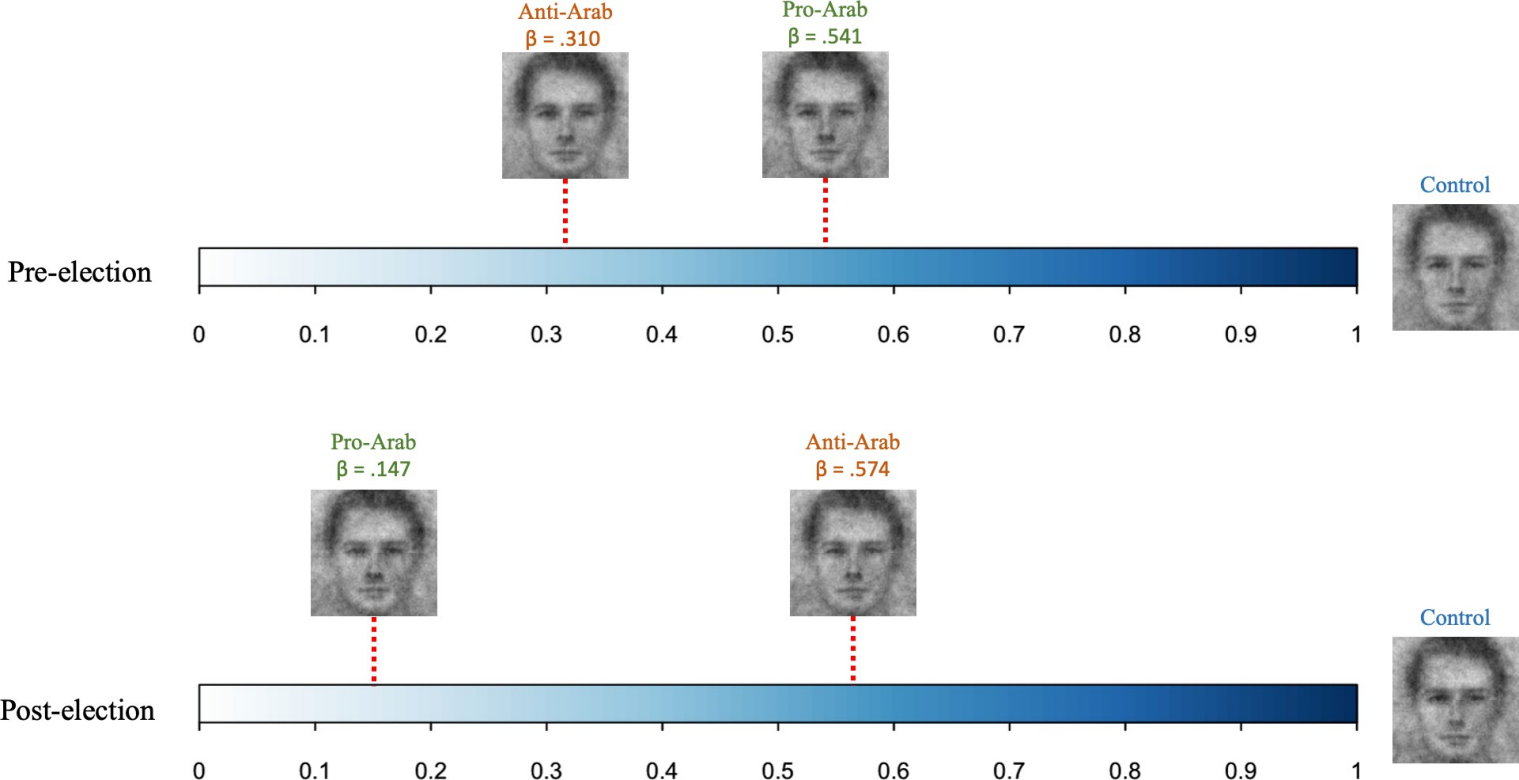
An example of changes in representational similarities (in beta values) among conditions from pre-election to post election (American faces).

We then predicted the trait representations of the post-election, no rhetoric condition American face with the linear combination of the trait representations of the post-election, pro-Arab condition American face trait rating data and the trait representations of the post-election, anti-Arab condition Arab face. We found that *after* the election the pro-Arab condition trait representation was no longer a significant predictor of the no rhetoric condition trait representation (β = .147, SE = .096, t = 1.04, p = .301), whereas the anti-Arab condition trait representation remained a significant predictor (β = .574, SE = .121, t = 406, p < .001). Linear hypothesis testing showed that the pro-Arab condition trait representation and the anti-Arab condition trait representation marginally differed in the extent to which they predicted the no rhetoric condition trait representation (F(1,75) = 3.54, p = .064). See Fig 4 for an illustration of these analyses.

#### Implicit attitudes

We used an Implicit Association Test [24, 25] to examine implicit attitudes that our participants expressed about Americans and Arabs. We conducted a three-way analysis of variance to test the effects of Rhetoric Type (pro-Arab, anti-Arab, no rhetoric), Site (USA, UAE), Time (pre-election, post-election), and all interactions among those three variables on our participants’ IAT scores. The results showed no main effect of Rhetoric Type, F(2,756) = 1.66, p = .191, η_p_^2^ < .001, which suggests that implicit attitudes were not sensitive to rhetoric exposure. The main effect of Site was significant, F(1,756) = 54.39, p < .001, η_p_^2^ = .067: across three conditions American participants showed significantly greater implicit ingroup preference (M = .55, SD = .60) than Arab participants (M = .23, SD = .59). The main effect of Time was not significant, F(1,756) = .03, p = .865, η_p_^2^ < .001. None of the interaction effects were significant.

Thus, on an implicit level, ingroup bias was the dominant response at all time points irrespective of the rhetoric condition. Although our Arab participants demonstrated consistent implicit ingroup favoritism, the magnitude of this effect was less than our American participants, which could reflect a relative implicit positivity toward US culture and cities or the national variability in the Arab locations used in our IAT.

### Explicit attitudes

To assess explicit attitudes, we examined our participants’ self-reported feeling thermometer evaluations of Americans and Arabs. For both the USA and UAE college students, their evaluations of outgroup (Arab and American, respectively) on an explicit level were positive and significantly above the neutral midpoint (i.e., 50) at all time points: American participants’ evaluation of Arab people (M = 68.30, SD = 21.85, t(380) = 16.35, p < .001) and Arab participants’ evaluation of American people (M = 58.42, SD = 21.26), t(326) = 7.16, p < .001, averaged across two time points. This further confirmed that our participants were among a segment of the population in their respective regions that explicitly viewed each other favorably.

We then conducted a three-way analysis of variance to test the effects of Rhetoric Type (pro-Arab, anti-Arab, no rhetoric), Site (USA, UAE), Time (pre-election, post-election), and all interactions among those three variables on our participants’ explicit attitude about Arabs and Americans. This analysis allowed us to probe whether, despite overall positive attitudes about each other, either group showed an explicit ingroup bias and whether such a bias was moderated by exposure to pro- or anti-Arab political rhetoric and the Trump administration gaining power and implementing anti-Muslim policies. The results showed no main effect of Rhetoric Type, F(2,688) = 1.06, p = .348, η_p_^2^ < .001 indicating that explicit attitude scores did not differ as a function of exposure to pro-Arab or anti-Arab political rhetoric. The main effect of Site was significant, F(1,688) = 28.52, p < .001, η_p_^2^ = .04: on average American participants showed significantly less explicit ingroup preference (M = -2.15, SD = 25.35) than Arab participants (M = 8.16, SD = 25.79). The main effect of time was marginally significant, F(1,688) = 2.91, p = .088, η_p_^2^ < .001 meaning that participants showed marginally less explicit ingroup bias after the election (M = 1.13, SD = 26.29) compared to before the election (M = 3.90, SD = 25.78). The only significant interaction effect was between Site and Time, F(1,688) = 8.06, p = .005, η_p_^2^ = .01. A follow-up Tukey’s HSD post-hoc test of this interaction effect revealed that before the election, Arab participants’ explicit ingroup preference scores (M = 6.81, SD = 26.60) did not statistically differ from those of American participants (M = 1.69, SD = 24.99), p = .227. However, after the election, Arab participants showed significantly greater explicit ingroup preference (M = 9.44, SD = 25.02) than American participants (M = -6.70, SD = 25.10), p < .001, who showed an explicit outgroup preference. These results suggested that enactment of Trump’s anti-Muslim policies was related to our American participants to report more favorable views of Arabs (outgroup) than Americans (ingroup). This resulted in only our Arab participants exhibiting an explicit ingroup preference after Trump’s election. Arab’s explicit attitudes toward Americans did not differ between before and after the election.

## Discussion

Donald Trump’s rise in American politics provided a unique opportunity to investigate the effects of political information permeating domestic political group boundaries and spreading across the globe. Our research investigated psychological effects of anti-Arab politics promulgated by Trump and like-minded politicians. To this end, we conducted experiments to examine how a news article that quoted an American politician disparaging the Arab World before and after the election that made Trump the president of the U.S. influenced the social perceptions and attitudes of American and Arab individuals.

In this research, we were interested in understanding how attitudes and perceptions of American and Arab people have of each other change in respond to anti-Arab rhetoric at different levels of information processing, using measures of visual representation as well as explicit and implicit attitudes. We explored whether their views would become more negative in response to anti-Arab rhetoric due to negative associations being activated, reject them due to conflicts with their ideology, or the rhetoric would have minimal impact because the “Arab” and “American” categories are not novel and were already firmly established. Our findings revealed no evidence of rhetoric influencing attitudes, consistent with one of our predictions that attitudes may be resistant to change. However, we observed a nuanced pattern of results as a function of Trump’s election and ensuing implementation of anti-Muslim policies in visual representations.

The noteworthy findings from the face categorization task were that, in the absence of any rhetoric, American participants’ default visual representation of a typical Arab person resemble their representation following exposure to anti-Arab rhetoric more after the election than before. This shift appears to be driven by changes in default representations and anti-Arab representations from pre- to post-election, rather than changes in pro-Arab representations. Similarly, Arab participants’ visual representations of a typical American person when not exposed to any rhetoric resemble their representation after reading the anti-Arab rhetoric more after the election than before the election. Once again, this shift was driven by changes in default and anti-Arab representations from pre- to post-election. It is crucial to note, however, that similarities in trait representations do not necessarily imply a more positive or negative evaluation. Instead, they suggest a more overall alignment in the pattern of ratings for both positive and negative traits. For example, relationships between traits (e.g., aggressive and trustworthy) have been shown to change across context and group boundaries [33, 34]. Thus, depending on the rhetoric and election conditions, the network of trait relationships might have shifted. Future research should delve deeper into understanding the dynamics of these shifts and their implications within the context of political rhetoric and policies. Overall, these face categorization findings suggest that both American and Arab participants’ visual representations of each other assimilated to anti-Arab rhetoric after the election. This indicates that the categorization stage was particularly sensitive to rhetoric and election effects, leading to anti-Arab politics being more accessible and salient in their minds after the election than before the election.

Both implicit and explicit attitude results were not affected by rhetoric, indicating that American and Arab people’s attitudes towards each other at both the characterization and correction stage are not swayed by the rhetoric. Implicit attitudes were likewise unaffected by the election results, with both Arab and American participants exhibiting implicit ingroup preference before and after the election. This is notable because it provides robust evidence that the IAT captures average intergroup attitudes and serves as a counterpoint to recent criticism of the usefulness of the IAT as an attitude measure [35]. In contrast to the implicit attitude results, the shift in American attitudes and policy reflected in Trump’s election and a subsequent travel ban of seven predominantly Muslim countries did have an impact on explicit attitudes. Prior to the election, American and Arab participants did not statistically differ in their explicit ingroup preference. After the election, Arab participants continued to show an explicit ingroup preference, whereas American participants continued to show an explicit outgroup preference. This finding suggests that election results may have influenced the correction stage for American participants, leading to patterns of results that is in line with the prediction that their attitudes toward Arab people might even become more positive after exposure to anti-Arab rhetoric. The lack of a correspondence between the implicit and explicit attitudes for Americans shows that even though Americans in a predominantly liberal state such as California might report no explicit bias or even relatively pro-Arab attitudes, at an implicit level they still hold pro-American biases. This result is consistent with evidence that explicit attitudes change more quickly in response to new information [36], and likely reflect a more controllable, ideologically-consistent response.

In summary, these results indicate that Americans electing a President who espoused anti-Muslim and anti-Arab rhetoric was related to psychological shifts in both American and Arab participants’ default mental representations of each other as they became more similar to when they read anti-Arab rhetoric post-election compared to pre-election. This was true even though implicit attitudes seemed insensitive to both rhetoric and election outcome and American participants expressed even more positive explicit attitudes of Arab people post-election.

This work provides a window into how American politics can influence the various levels of information processing that guide how Americans and Middle Eastern Arabs view each other, and in doing so, reflects calls to include non-Western participants in psychological research [37, 38]. However, it is important to emphasize that our results specifically apply to people in both regions who may have a favorable view of each other given that our Americans were from a predominantly liberal state and the UAE is a political ally of the United States. We also had greater numbers of American participants than Arab participants, further limiting generalizability of our work. It is unclear whether right wing Americans who endorse Trump’s nativist views or people in the Arab Middle East who despise the USA would show a similar pattern of results. Both the USA and the Arab Middle East are diverse and complex, so it would be a mistake to overgeneralize from our California and UAE-based student participants. Moreover, although the measures we used are some of the most frequent assessments in the social cognition literature and have been validated in several contexts [20, 39], recent methodological criticisms [35, 40] suggest that future research should examine if similar results emerge with other techniques [41]. Additionally, our inferences about the effects of the election are susceptible to problems inherent with correlational research. For instance, it is unknown whether differences in world events that co-occurred with our pre- and post-election measurement periods could have contributed to our election effects, nor are we certain whether it was the election itself–and the fact that millions of Americans voted for Trump–or the enactment of discriminatory policies against Muslims (an identity also held by a large percentage of our Arab participants) that account for our effects. Future work should examine how much our findings generalize to a broader swath of American and Arab participants in subsequent elections and use additional psychological measures to test the robustness of our conclusions.

What is incontrovertible, however, is that in today’s interconnected world, American politics reverberate across the country and throughout the world. Our research demonstrates the value of embedding cross-national experimental manipulations within real-world quasi-experimental designs and going beyond conventional self-report assessment to include social cognitive measures that probe multiple stages of information processing. Although we focused our investigation on rhetoric and policies about Arabs in American political discourse, other groups (e.g., Latinx, African, Haitian, Russian, and Chinese people) have also been targeted in recent years. The cumulative psychological effects of these hardline politics against various groups are unknown. Our findings suggest that such political rhetoric and policies might not change impressions and evaluations as much as conventional wisdom assumes, but it is important to consider nuanced effects on various information processing stages when investigating cross-national social perception.
